# Case report: Findings of automated perimetry during a migraine episode in a patient with glaucoma

**DOI:** 10.3389/fmed.2022.950148

**Published:** 2022-10-28

**Authors:** Shunsuke Nakakura, Satomi Oogi, Asaya Tanoue, Teruyuki Miyoshi

**Affiliations:** ^1^Department of Ophthalmology, Saneikai Tsukazaki Hospital, Himeji, Japan; ^2^Miyoshi Eye Clinic, Department of Ophthalmology, Fukuyama, Japan

**Keywords:** automated perimetry, migraine, glaucoma, patient, case report, homonymous hemianopia

## Abstract

Comorbidities like glaucoma and migraine are often observed among middle-aged individuals, especially women. Herein, we report a rare case of a patient who underwent automated perimetry during a migraine attack. A 52-year-old woman with a 1-year history of blurred vision in the nasal field of her right eye visited Miyoshi Eye Clinic. The intraocular pressures of the right and left eyes were 22 and 24 mm Hg, respectively. Retinal imaging revealed a retinal nerve fiber defect in the temporal superior macula with corresponding thinning of the superior ganglion cell complex in the right eye. The left eye appeared normal. Primary open-angle glaucoma was suspected, and the patient underwent a visual field examination on the same day. Perimetry showed that the mean deviations in the right and left eyes were −5.00 and −7.68 dB, respectively. A visual field defect in the inferior nasal aspect of the right eye corresponded to the retinal nerve fiber defect. However, right-sided homonymous hemianopia–like visual field defects were observed in both eyes. After the examination, the patient stated that a migraine attack had started 5 min before the examination and continued till after its end (attack duration was ∼20 min). In the follow-up examinations without migraine, homonymous hemianopia-like visual field defects disappeared, and only a glaucomatous visual field defect in the right eye was observed. Hence, the initial visual field examination findings reflected the effects of a migraine attack alongside glaucoma. Detailed interviews with patients may be beneficial for understanding visual field findings and preventing their untimely examination.

## Introduction

Migraine, a common neurological headache disorder, affects 10–15% of individuals worldwide, especially those of working age (1). Typical migraines are characterized by headache; nausea, vomiting, or both; photophobia and phonophobia; and mild blurring of vision (2). Glaucoma is also a common ocular disease whose prevalence increases with age. In 2020, glaucoma was responsible for 11% of all cases of blindness globally in adults aged ≥ 50 years (3). Some studies (4–6) have suggested that migraine increases the risk of developing glaucoma. However, there have been studies reporting no such findings (7, 8). Hence, the relationship between migraine and glaucoma is yet to be fully clarified. Visual field defects are characteristic of glaucoma and are also experienced by patients with migraine (9–17); furthermore, migraine and glaucoma often present as comorbidities. Here we report a case of primary open-angle glaucoma wherein automated perimetry during a migraine episode revealed unique visual field defects.

## Case description

This case report was approved by the Institutional Review Board of Saneikai Tsukazaki Hospital, Himeji, Japan (No. 221002). All examinations were conducted according to the Declaration of Helsinki.

A 52-year-old woman visited Miyoshi Eye Clinic in Fukuyama, Japan, with complaints of blurred vision in the nasal field of her right eye persisting for 1 year. Visual acuities and intraocular pressures (according to Goldmann applanation tonometry) in the right and left eyes of the patient were 0.2 (1.0 × S–2.25D) and 0.2 (1.0 × S–2.75D) and 22 and 24 mm Hg, respectively.

**FIGURE 1:**
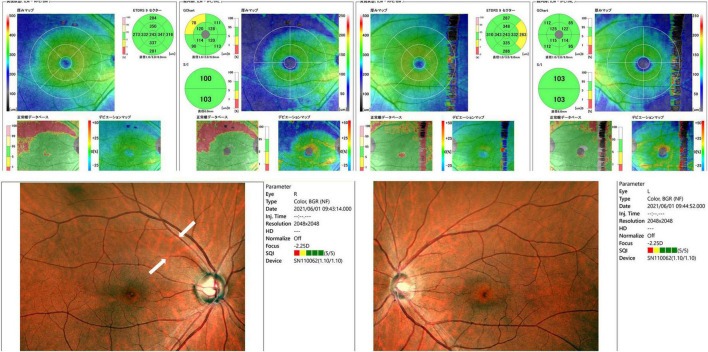
Retinal photographs and thickness evaluation of the macular ganglion cell complex using optical coherence tomography. Retinal nerve fiber layer defects (white arrows, bottom left image) were observed in the right eye. Optical coherence tomography also showed thinning of the macular ganglion cell complex in the temporal superior aspect.

**FIGURE 2:**
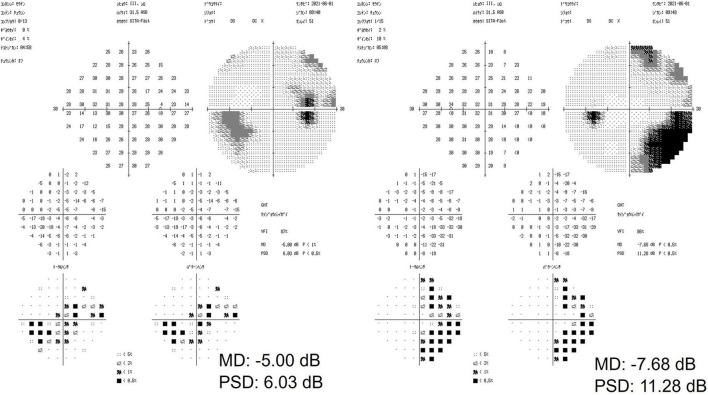
The results of a visual field examination using a Humphrey field analyzer. In the right eye, a visual field defect in the nasal inferior aspect corresponded to a defect in the retinal nerve fiber layer. In both eyes, a right-sided homonymous hemianopia-like visual field defect was observed.

Slit-lamp examination revealed no inflammation in both eyes, which exhibited normal anterior chamber depth. A Mirante Scanning Laser Ophthalmoscope (Nideck Co., Gamagori, Japan) was employed to capture retinal photographs. Additionally, the RS-3000 system of optical coherence tomography (Nideck Co.) was used to measure the thickness of the macular ganglion cell complex (Figure 1). The color image of the right eye revealed a defect of the retinal nerve fiber layer and a corresponding notch sign in the superior optic disk. Moreover, optical coherence tomography demonstrated thinning of the macular ganglion cell complex in the temporal superior aspect (Figure 1, left panel). The left eye of the patient exhibited no apparent glaucomatous alterations (Figure 1, right panel). The findings of the right eye indicated primary open-angle glaucoma, and a visual field examination using a Humphrey field analyzer (HFA; Carl Zeiss Meditec AG, Dublin, CA; 30-2 Swedish Interactive Threshold Algorithm standard) was performed on the same day. HFA was conducted from the right to the left eye without interval. The mean deviations of the right and left eyes were −5.00 and −7.68 dB, respectively (Figure 2). A visual field defect in the nasal inferior aspect of the right eye corresponded to the retinal nerve fiber layer defect observed earlier. However, in both eyes, a right-sided homonymous hemianopia-like visual field defect was observed in both grayscale and pattern deviation images (Figure 2). Following perimetry, the patient stated that she experienced a migraine attack with an aura during the HFA examination. Consequently, a detailed medical interview regarding the migraine history of the patient was conducted.

The patient started experiencing migraines at the age of 17 years, with a frequency of several times per month. However, the frequency of the attacks and the headache during migraine attack decreased with age. Presently, the migraine symptom of the patient involved a visual disturbance aura almost without headache. She described the visual disturbance as dazzling white areas that emerged at various points in her visual field and spread across the whole field in a wave. The direction of the waves varied with each attack. The migraine aura and headache alternated between the right and left visual fields and frontal, lateral, or both hemispheres, respectively. As the patient did not exhibit vertigo or dizziness (18), her migraine was categorized as ICHD-code 1.2.1.2 “Typical aura without headache” (19).

The visual disturbance started 5 min before the HFA examination. The patient expressed that she was nervous during the examination. During the examination, the patient developed the visual disturbance. The migraine attack intensity peaked following the completion of the HFA examination of both eyes, and the attack lasted for ∼20 min.

The patient was started on antiglaucoma medication to prevent glaucoma progression. The intraocular pressure of the patient was controlled at approximately 18–20 mm Hg at the 11-month follow-up.

**FIGURE 3:**
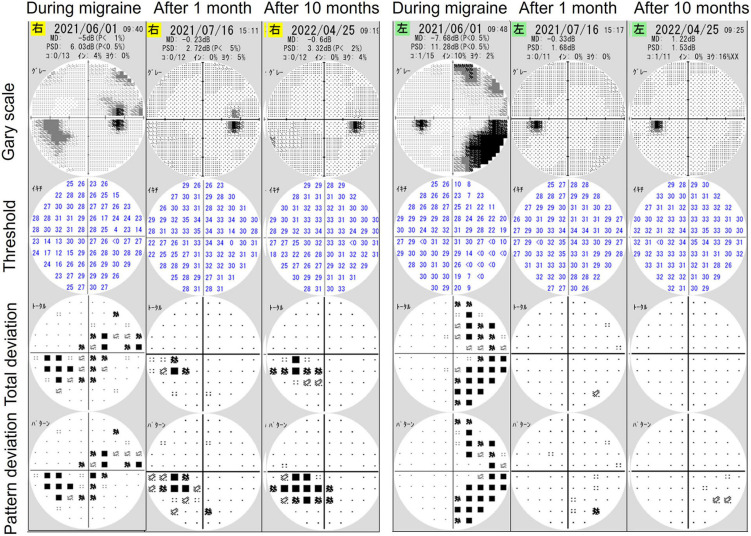
The results of visual field examinations using a Humphrey field analyzer (HFA) 1 and 10 months after initial HFA examination during a migraine attack. Follow-up findings revealed only glaucomatous visual field defects in the right eye without right-sided homonymous hemianopia-like visual field defects.

The HFA examination was repeated 1 and 10 months following the first examination (Figure 3), and mean deviations of −0.23 and −0.60 dB and −0.33 and 1.22 dB of the right and left eyes, respectively, were observed. During both examinations, the patient exhibited no migraine symptoms. The examination findings indicated no homonymous hemianopia–like visual field defects. The only abnormality was the visual field defect in the nasal inferior aspect of the right eye, corresponding to the retinal nerve fiber layer defect (Figure 3). Hence, the findings of the first HFA examination, which was performed during a migraine attack, reflected the effects of both glaucoma and migraine aura on the visual field.

## Discussion

Here we reported a case of migraine-related visual field defect observed during perimetry in a patient with typical glaucoma. Luu et al. (9) reported a case of right-sided homonymous hemianopia assessed using HFA during a migraine attack; the HFA findings were similar to those of our patient. Furthermore, Yohannan and Jampel (10) reported a case of a left inferior quadrantanopsia using HFA during a migraine attack in a patient suspected of glaucoma. Both studies reported that repeated perimetry showed a resolution of anopia and a normal visual field (9, 10).

Yener and Korucu (11) compared HFA findings during attacks both in patients with migraine without aura and in those with tension-type headache and found no difference in their mean and pattern deviation values. Furthermore, the patients reportedly exhibited different patterns of non-specific visual field defects (11). Using automated perimetry (Dremel DigiLab 750; BioRad Laboratories, Hercules, CA, USA), Ebner (17) reported visual field depression, except in the central 5°, in a patient with homonymous type distribution during a migraine attack. During such an episode, aura, headache, and a consequent decrease in the ability to concentrate may impact visual field findings. Migraine is considered to have a cortical origin because visual auras are homonymous and hemianopic (2). Visual auras comprise transient neurological disturbances of sight (90% of cases), disturbances of speech, or tingling/numbness of the face or body (2). Other visual field analyses using automated perimetry following a migraine attack have been reported (12–16). In these reports, a week after the attack, a decrease only in the sensitivity of the examination was observed (12–15). Additionally, a bilateral homonymous deficit was not observed in any case (12–15). Therefore, homonymous hemianopia visual field defect (9, 10, 17) can be considered as a common visual field characteristic of migraine attacks. Furthermore, this supports our HFA examination findings during a migraine attack. However, whether the homonymous hemianopia visual field defect reflects the ongoing visual auras or transient cerebral cortex paralysis remains unknown. A study reported that decreased sensitivity following the headache lasts 30–40 days on average, with a few cases showing durations of up to 75 days (16). Fortunately, the visual field defects in our patient recovered after 1 month. However, ophthalmologists should consider migraine episodes while evaluating the visual fields of patients with glaucoma.

## Conclusion

We described the findings of an automated visual field examination performed on a patient with typical glaucoma during a migraine attack. Detailed interviews with patients may be beneficial for understanding visual field findings and preventing their untimely examination.

## Data availability statement

Data are available upon reasonable request. All data relevant to the study are accessed by the corresponding author. No additional data are available.

## Ethics statement

The studies involving human participants were reviewed and approved by the Institutional Review Board of Saneikai Tsukazaki Hospital, Himeji, Japan (No. 221002). The patients/participants provided their written informed consent to participate in this study.

## Author contributions

SN: data collection, study design and interpretation, manuscript drafting, and figures creation. SO: data collection, manuscript review, and editing. AT and TM: data collection and manuscript review. All authors contributed to the article and approved the submitted version.

## References

[1] Stovner LJ Hagen K Jensen R Katsarava Z Lipton R Scher A The global burden of headache: a documentation of headache prevalence and disability worldwide. *Cephalalgia.* (2007) 27:193–210. 10.1111/j.1468-2982.2007.01288.x 17381554

[2] Nguyen BN Lek JJ Vingrys AJ McKendrick AM. Clinical impact of migraine for the management of glaucoma patients. *Prog Retin Eye Res.* (2016) 51:107–24. 10.1016/j.preteyeres.2015.07.006 26232725

[3] Gbd 2019 Blindness and Vision Impairment Collaborators, Vision Loss Expert Group of the Global Burden of Disease Study. Causes of blindness and vision impairment in 2020 and trends over 30 years, and prevalence of avoidable blindness in relation to VISION 2020: the Right to Sight: an analysis for the global burden of disease study. *Lancet Glob Health.* (2021) 9:e144–60. 10.1016/S2214-109X(20)30489-7 33275949 PMC7820391

[4] Huang JY Su CC Wang TH Tsai IJ. Migraine and increased risk of developing open angle glaucoma: a population-based cohort study. *BMC Ophthalmol.* (2019) 19:50. 10.1186/s12886-019-1062-9 30760249 PMC6375150

[5] Cursiefen C Wisse M Cursiefen S Jünemann A Martus P Korth M. Migraine and tension headache in high-pressure and normal-pressure glaucoma. *Am J Ophthalmol.* (2000) 129:102–4. 10.1016/s0002-9394(99)00289-5 10653426

[6] Funk RO Hodge DO Kohli D Roddy GW. Multiple systemic vascular risk factors are associated with low-tension glaucoma. *J Glaucoma.* (2022) 31:15–22. 10.1097/IJG.0000000000001964 34731871 PMC9337264

[7] Usui T Iwata K Shirakashi M Abe H. Prevalence of migraine in low-tension glaucoma and primary open-angle glaucoma in Japanese. *Br J Ophthalmol.* (1991) 75:224–6. 10.1136/bjo.75.4.224 2021590 PMC1042327

[8] Chen HY Lin CL Kao CH. Does migraine increase the risk of glaucoma?: a population-based cohort study. *Medicine.* (2016) 95:e3670. 10.1097/MD.0000000000003670 27175700 PMC4902542

[9] Luu ST Pesudovs K Lee AW Chen CS. Homonymous hemianopia captured on automated perimetry during a migraine episode. *Intern Med J.* (2010) 40:310–1. 10.1111/j.1445-5994.2010.02194.x 20180871

[10] Yohannan J Jampel H. Progressing scintillating scotoma captured on automated visual field testing. *Ophthalmology.* (2016) 123:1395–6. 10.1016/j.ophtha.2016.01.002 26854039

[11] Yener AÜ Korucu O. Visual field losses in patients with migraine without aura and tension-type headache. *Neuroophthalmology.* (2017) 41:59–67. 10.1080/01658107.2016.1251466 28348627 PMC5354103

[12] Comoğlu S Yarangümeli A Köz OG Elhan AH Kural G. Glaucomatous visual field defects in patients with migraine. *J Neurol.* (2003) 250:201–6. 10.1007/s00415-003-0975-6 12574951

[13] Yenice O Temel A Incili B Tuncer N. Short-wavelength automated perimetry in patients with migraine. *Graefes Arch Clin Exp Ophthalmol.* (2006) 244:589–95. 10.1007/s00417-005-0083-7 16175372

[14] McKendrick AM Vingrys AJ Badcock DR Heywood JT. Visual dysfunction between migraine events. *Invest Ophthalmol Vis Sci.* (2001) 42:626–33. 11222520

[15] McKendrick AM Badcock DR. Decreased visual field sensitivity measured 1 day, then 1 week, after migraine. *Invest Ophthalmol Vis Sci.* (2004) 45:1061–70. 10.1167/iovs.03-0936 15037569

[16] McKendrick AM Vingrys AJ Badcock DR Heywood JT. Visual field losses in subjects with migraine headaches. *Invest Ophthalmol Vis Sci.* (2000) 41:1239–47. 10752965

[17] Ebner R. Visual field examination during transient migrainous visual loss. *J Clin Neuroophthalmol.* (1991) 11:114–7. 1832684

[18] Nowaczewska M. Vestibular migraine - an underdiagnosed cause of vertigo. Diagnosis and treatment. *Neurol Neurochir Pol.* (2020) 54:106–15. 10.1590/0004-282X20160037 32285435

[19] Cephalalgia,. Headache classification committee of the international headache society (IHS) The international classification of headache disorders, 3rd edition. *Cephalalgia.* (2018) 38:1–211. 10.1177/0333102417738202 29368949

